# Combined associations of tea consumption and smoking with accelerated biological aging among oilfield workers

**DOI:** 10.3389/fnut.2026.1762577

**Published:** 2026-06-03

**Authors:** Bowei Yang, Haobiao Liu, Xuefeng Yu, Licheng Yang, Qingsong Li, Jing Tang, Yingjie Cai, Abebe Feyissa Amhare, Ziwei Guo, Zhiyong Du, Jun Zhang, Jing Han

**Affiliations:** 1 Ningxia Gem Flower Hospital, Yinchuan, Ningxia, China; 2 School of Public Health, Health Science Center, Xi'an Jiaotong University, Xi'an, Shaanxi, China; 3 Department of Comprehensive Orthopedics, The Second Affiliated Hospital of Xi'an Jiaotong University, Xi'an, Shaanxi, China; 4 Xi'an Gem Flower Changqing Hospital, Xi'an, Shaanxi, China

**Keywords:** accelerated biological aging, cigarette smoking, occupational population, oilfield worker, tea consumption

## Abstract

**Objectives:**

This study aimed to examine the individual and combined associations of tea consumption and cigarette smoking with accelerated biological aging among oilfield workers.

**Methods:**

Logistic regression was used to assess the associations of tea consumption and cigarette smoking with accelerated biological aging, defined using the PhenoAge algorithm, with results reported as odds ratios (ORs) and 95% confidence intervals (CIs). Interaction and stratified analyses by shift work status were performed to evaluate additive, multiplicative, and context-specific effects. Sensitivity analyses were conducted to assess the robustness of the observed associations.

**Results:**

High-level tea consumption was associated with increased odds of accelerated aging (*OR* = 1.35, 95% *CI*: 1.06–1.72), whereas low-level or binary tea intake showed no significant effect. Cigarette smoking was strongly linked to accelerated aging (*OR* = 1.81, 95% *CI*: 1.42–2.31). In combined analyses, smokers with low or high tea intake had greater odds compared with non-smokers who did not drink tea (low-level: *OR* = 2.12, 95% *CI*: 1.49–3.01; high-level: *OR* = 2.29, 95% *CI*: 1.64–3.21), while tea alone among non-smokers was not significant. Stratified analyses by shift work indicated stronger combined effects among non-shift workers. Interaction analyses showed a modest additive interaction (RERI = 0.11, AP = 0.09, *S* = 2.33) and evidence of multiplicative interaction (*P* = 0.025).

**Conclusions:**

As the first study to investigate the joint effects of tea consumption and cigarette smoking on accelerated biological aging, our analysis revealed that tea consumption may amplify the aging-related risk associated with smoking. These findings highlight the importance of integrated nutritional and behavioral strategies to mitigate accelerated biological aging in occupational populations.

## 1 Introduction

The global population is aging at an unprecedented pace, bringing profound challenges for public health and workforce sustainability. With increased longevity comes a growing burden of age-related morbidities and functional decline. Traditionally, aging has been defined by chronological years, but individuals of the same age often differ markedly in the integrity of physiological systems that sustain health and resilience (1). This recognition has shifted attention from merely counting years to understanding the rate at which biological functions deteriorate—a trajectory that varies across individuals and determines susceptibility to chronic disease, frailty, and premature mortality (2, 3). These insights have prompted growing interest in identifying modifiable factors that may influence the pace of physiological decline.

Diet and lifestyle are modifiable factors that shape metabolism, inflammation, and the resilience of cellular systems, and as such they present opportunities to influence the trajectory of physiological change. In this regard, tea consumption is worthy of attention as a common dietary exposure with bioactive compounds and global reach. Several cohort analyses have associated habitual tea drinking with lower risk of cardiovascular disease, chronic kidney disease, and cognitive decline (4–6), and a large meta-analysis of prospective observational studies found daily tea consumption to be linked with reduced all-cause and cardiovascular mortality (4). At the same time, other large observational investigations have reported null associations or inconsistent results depending on tea variety or consumption patterns (7). When it comes to age-related physiological measures, some longitudinal studies suggest that tea drinking correlates with attenuation of biological aging and reduced likelihood of frailty transition (8, 9), yet others do not detect a clear protective effect (10). Moreover, in certain settings tea consumption has shown a positive association with metabolic syndrome (11), and a U-shaped relationship between tea intake and mortality has also been observed (12). This pattern—benefit in some groups, null or mixed in others—suggests that the association of tea as a dietary exposure with the rate of physiological decline is far from settled and may depend on context.

In contrast to the ambiguity surrounding tea, cigarette smoking is robustly associated with accelerated physiological decline. A large and consistent body of literature documents that smoking increases systemic oxidative stress and inflammatory mediators, perturbs proteostasis, and correlates with epigenetic signatures and telomere shortening that parallel an advanced physiological state (13, 14). Epigenome-wide studies repeatedly demonstrate that smoking correlates with accelerated epigenetic age across multiple tissues, and telomere studies similarly report shorter telomeres among smokers, although effect sizes vary and some tissue-specific patterns exist (15–17). Observational studies further support these associations in diverse populations. In U.S. middle-aged and older adults, current smoking is inversely associated with circulating levels of α-Klotho, an anti-aging protein (18). Moreover, prenatal exposure to maternal smoking is linked to shorter adult telomere length in offspring, with smoking during pregnancy associated with approximately 0.8% reduction in telomere length and a significant correlation with accelerated physiological aging (19). Collectively, these findings position smoking as one of the most robust behavioral predictors of adverse aging-related physiological markers and clinical outcomes.

Despite extensive work on tea and smoking separately, their joint presence has received little direct attention in relation to composite physiological indices. Behaviors such as dietary patterns and smoking often co-occur, and tea and tobacco share overlapping biochemical and pharmacological pathways. Both introduce exogenous compounds that affect oxidative metabolism and xenobiotic processing and can influence sympathetic activity and sleep patterns to varying degrees. It is therefore conceivable that tea intake could modify, amplify, or in some circumstances mask the physiological signatures associated with smoking, and vice versa. Moreover, the overall associations observed in epidemiological studies may depend on whether tea intake functions primarily as a nutrient-rich dietary exposure or as a compensatory behavior linked to other risk exposures.

Occupational groups provide a particularly informative setting in which to interrogate such joint effects. Workers in oil extraction and production are routinely exposed to irregular schedules, including night and rotating shifts, and to physical and chemical stressors such as noise, dust, hydrocarbon solvents, and intermittent high workload. These exposures can disrupt circadian rhythms, inflammatory homeostasis, and metabolic regulation, creating conditions in which lifestyle factors may have distinct or amplified consequences. Moreover, workplace routines shape the contexts in which beverages and tobacco are consumed, so that tea and smoking may co-occur within similar temporal or social patterns. Thus, an investigation among oil-field workers can both clarify whether tea's putative nutritional effects translate into occupational settings and reveal whether concomitant smoking modifies any association with accelerated deviation from expected physiological state.

Against this background, the present study investigates habitual tea consumption and smoking among oilfield workers, focusing on their individual and combined associations with accelerated biological aging. By examining whether tea and smoking act independently, synergistically, or antagonistically, this research aims to provide evidence that can inform dietary guidance and workplace health strategies that consider the combined effects of dietary and lifestyle behaviors in occupational settings.

## 2 Materials and methods

### 2.1 Study population

**Figure 1:**
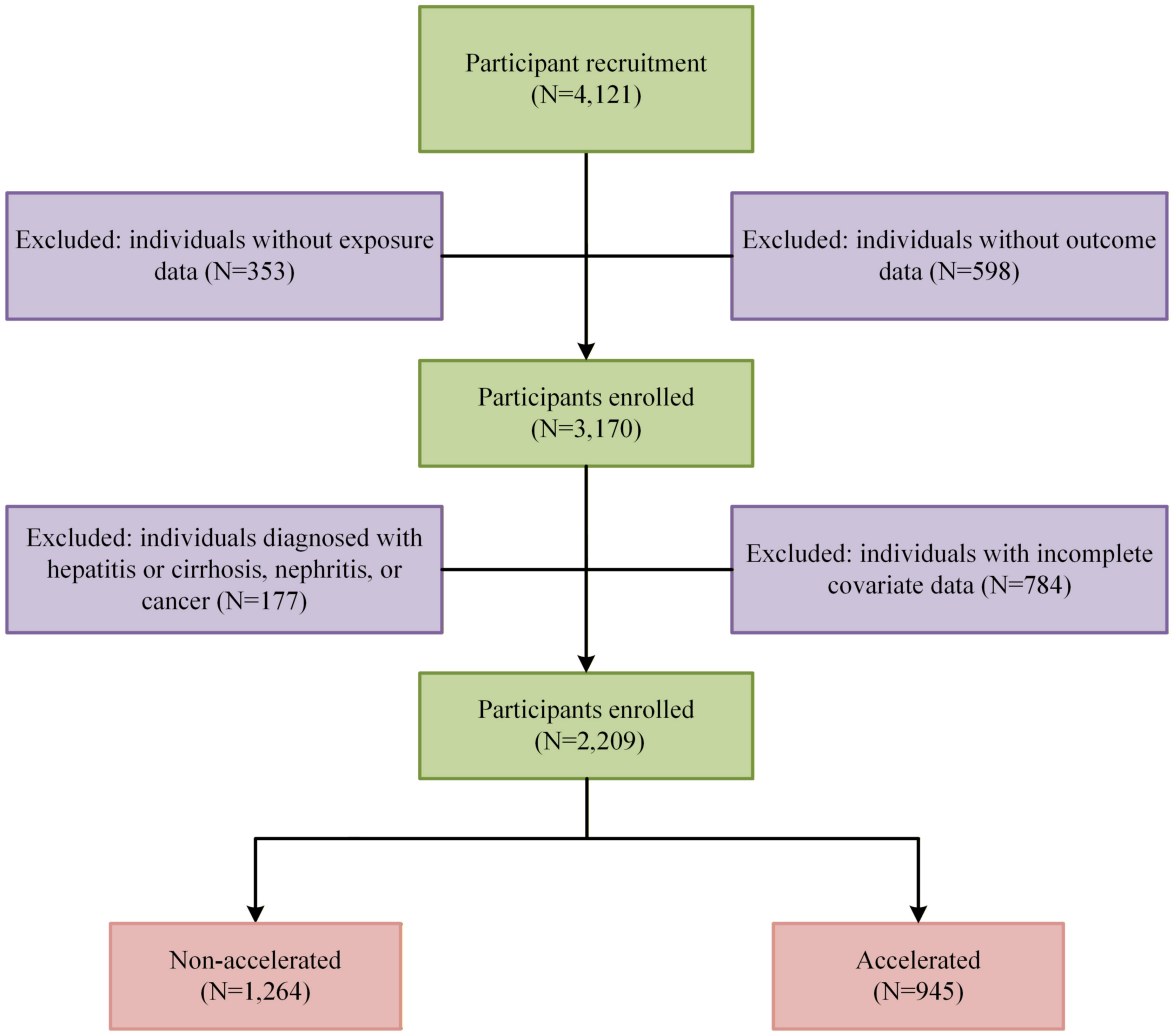
Flow diagram of participants included in this study.

This cross-sectional study was conducted among oilfield workers in Xi'an, China. Detailed information regarding the study design has been described elsewhere (11). A total of 4,121 workers were initially recruited. Among them, 353 individuals lacked exposure data on tea consumption or smoking, 598 had missing outcome information, and 177 were excluded due to physician-diagnosed liver diseases (hepatitis or cirrhosis), nephritis, or cancer. These conditions were excluded *a priori* because they can substantially alter inflammatory, metabolic, and hematological biomarkers used to derive phenotypic age. In addition, 784 participants had incomplete covariate data. After applying these exclusion criteria, 2,209 participants were included in the final analysis (Figure 1).

### 2.2 Assessment of exposures

Information on tea consumption was collected using a structured questionnaire administered by trained investigators. Participants were asked whether they usually drank tea, defined as drinking tea at least three times per week for a duration of 6 months or longer. Those who answered “yes” were further asked about their weekly frequency and the average amount consumed per occasion. Investigators provided a 500-ml cup as a visual reference to help participants estimate the quantity consumed. Weekly tea intake (mL/week) was calculated by multiplying the reported frequency by the amount consumed each time. Participants were then categorized into three groups—non-drinkers, low-level drinkers, and high-level drinkers—using the median value of tea intake among drinkers as the cutoff.

Smoking behavior was assessed through standardized interview questions. Participants were asked whether they smoked cigarettes, defined as smoking at least one cigarette per day for a duration of 6 months or longer. Those who met this criterion were classified as smokers, while others were classified as non-smokers.

To examine the combined effects of tea drinking and smoking, participants were categorized according to their exposure status. Participants were divided into four mutually exclusive groups according to tea drinking and smoking status: non-tea drinkers and non-smokers, tea drinkers only, smokers only, and tea drinkers and smokers. To further explore dose-response patterns, a six-group classification was constructed by combining tea-drinking levels (non-drinkers, low-level drinkers, and high-level drinkers) with smoking status.

### 2.3 Assessment of outcome

The primary outcome was phenotypic age acceleration, a measure of biological aging derived from the difference between an individual's estimated phenotypic age and their chronological age. Phenotypic age was estimated using the PhenoAge algorithm developed by Levine and colleagues (20), a validated clinical aging metric designed to capture age-related physiological deterioration and mortality risk. Specifically, PhenoAge was calculated based on a weighted algorithm incorporating chronological age and nine routine measured clinical biomarkers: albumin, creatinine, glucose, C-reactive protein, lymphocyte percentage, mean cell volume, red cell distribution width, alkaline phosphatase, and white blood cell count. This algorithm reflects a phenotypic dimension of biological aging, emphasizing systemic inflammation, metabolic function, and hematological integrity, rather than molecular or epigenetic aging processes. Phenotypic age acceleration was defined as the residual from a linear regression model of phenotypic age on chronological age. A positive residual indicates that an individual's physiological status is older than expected for their chronological age. A negative residual suggests a younger-than-expected physiological profile. For categorical analyses, we classified participants into two groups based on whether their phenotypic age acceleration value exceeded zero.

### 2.4 Covariates

Potential confounders were selected based on prior literature and biological plausibility (11, 21, 22). Covariates included sex, ethnicity, education level, marital status, annual income, body mass index, alcohol drinking, physical activity, shift work, chemical substance exposure, noise exposure, dust exposure, salt intake, food diversity, hypertension, hyperlipidemia, and cardiovascular disease. Detailed information regarding covariates can be found in Supplementary Table 1.

### 2.5 Statistical analysis

Baseline characteristics of participants were summarized according to categories of phenotypic age acceleration. Categorical variables were expressed as frequencies and percentages, while continuous variables were presented as means with standard deviations. Differences between groups were assessed using the chi-square test for categorical variables and the Student's *t*-test for continuous variables.

Logistic regression models were applied to estimate odds ratios (ORs) and 95% confidence intervals (CIs) for the associations between tea consumption, smoking, and accelerated biological aging. Three progressively adjusted models were developed. The Model 1 included no covariate adjustment. Model 2 adjusted for sex, ethnicity, education level, marital status, and annual income. Model 3 additionally adjusted for body mass index, alcohol drinking, physical activity, shift work, chemical substance exposure, noise exposure, dust exposure, salt intake, food diversity, hypertension, hyperlipidemia, and cardiovascular disease. To further address potential behavioral confounding by smoking characteristics, we conducted stratified analyses by smoking status. Within smokers and non-smokers separately, we examined the association between tea consumption level and accelerated biological aging using multivariable logistic regression models.

Interaction analyses were conducted to examine potential additive and multiplicative effects between tea consumption and smoking. Additive interaction was evaluated by calculating three indices: the relative excess risk due to interaction (RERI), the attributable proportion due to interaction (AP), and the synergy index (*S*). A RERI or AP greater than zero, or an *S* greater than one, was considered evidence of a positive additive interaction. Multiplicative interaction was assessed by adding a cross-product term of tea consumption and smoking to the logistic regression model, and its statistical significance was tested using the Wald chi-square statistic.

Stratified analyses were further performed by shift work status, as shift schedules can affect circadian rhythms, oxidative stress, and metabolic regulation, thereby potentially modifying the association between lifestyle factors and biological aging. Several sensitivity analyses were conducted to evaluate the robustness of the findings, including treating phenotypic age acceleration as a continuous outcome, redefining the dichotomous cutoff using the median value, repeating all models after imputing missing covariate data using multiple imputation by chained equations, excluding participants with extreme biomarker values that exceeded three standard deviations from the mean, and using a refined classification of smoking status. All analyses were performed using R software (version 4.4.0), and statistical significance was defined as a two-sided *P* < 0.05.

## 3 Result

### 3.1 Baseline characteristics of participants

A total of 2,209 participants were included in the present analysis, with a mean age of 40.93 years, and 65.14% were males. Table 1 presents the baseline characteristics according to biological aging status. Compared with the non-accelerated group, participants with accelerated biological aging were more likely to be male, unmarried/separated, and obese. They also had higher proportions of cigarette smoking, alcohol drinking, and tea consumption, as well as higher prevalences of hypertension and hyperlipidemia. Participants in the accelerated aging group were more likely to report higher annual income and dust exposure, but were less likely to engage in shift work. No significant group differences were observed for age, ethnicity, education level, chemical substance exposure, noise exposure, physical activity, salt intake, food diversity, or cardiovascular disease.

**Table 1:** Baseline characteristics of participants by biological aging status.

Variables	Total (*N* = 2,209)	Non-accelerated (*N* = 1,264)	Accelerated (*N* = 945)	*P* value
Age, year	40.93 (8.30)	41.01 (7.90)	40.83 (8.81)	0.609
Sex				
Male	1,439 (65.14%)	708 (56.01%)	731 (77.35%)	<0.001
Female	770 (34.86%)	556 (43.99%)	214 (22.65%)	
Ethnicity				
Han	2,164 (97.96%)	1,236 (97.78%)	928 (98.20%)	0.594
Other	45 (2.04%)	28 (2.22%)	17 (1.80%)	
Education level				
High school or below	791 (35.81%)	446 (35.28%)	345 (36.51%)	0.821
College degree	808 (36.58%)	468 (37.03%)	340 (35.98%)	
University graduate or above	610 (27.61%)	350 (27.69%)	260 (27.51%)	
Marital status				
Married	1,863 (84.34%)	1,087 (86.00%)	776 (82.12%)	0.015
Unmarried/separated	346 (15.66%)	177 (14.00%)	169 (17.88%)	
Annual income, thousand (RMB)				
≤ 100	425 (19.24%)	243 (19.22%)	182 (19.26%)	0.017
101–150	1,355 (61.34%)	801 (63.37%)	554 (58.62%)	
≥151	429 (19.42%)	220 (17.41%)	209 (22.12%)	
Body mass index				
Underweight/normal	1,056 (47.83%)	730 (57.80%)	326 (34.50%)	<0.001
Overweight	799 (36.19%)	406 (32.15%)	393 (41.59%)	
Obesity	353 (15.99%)	127 (10.06%)	226 (23.92%)	
Shift work				
No	644 (29.15%)	336 (26.58%)	308 (32.59%)	0.002
Yes	1,565 (70.85%)	928 (73.42%)	637 (67.41%)	
Chemical substance exposure				
No	444 (20.10%)	244 (19.30%)	200 (21.16%)	0.305
Yes	1,765 (79.90%)	1,020 (80.70%)	745 (78.84%)	
Noise exposure				
No	838 (37.94%)	478 (37.82%)	360 (38.10%)	0.929
Yes	1,371 (62.06%)	786 (62.18%)	585 (61.90%)	
Dust exposure				
No	1,675 (75.83%)	981 (77.61%)	694 (73.44%)	0.027
Yes	534 (24.17%)	283 (22.39%)	251 (26.56%)	
Cigarette smoking				
No	1,196 (54.14%)	807 (63.84%)	389 (41.16%)	<0.001
Yes	1,013 (45.86%)	457 (36.16%)	556 (58.84%)	
Alcohol drinking				
No	1,368 (61.93%)	848 (67.09%)	520 (55.03%)	<0.001
Yes	841 (38.07%)	416 (32.91%)	425 (44.97%)	
Tea drinking				
No	1,061 (48.03%)	673 (53.24%)	388 (41.06%)	<0.001
Yes	1,148 (51.97%)	591 (46.76%)	557 (58.94%)	
Inactive	492 (22.27%)	289 (22.86%)	203 (21.48%)	0.471
Active	1,717 (77.73%)	975 (77.14%)	742 (78.52%)	
Salt intake				
≤ 6 g/day	1,081 (48.94%)	636 (50.32%)	445 (47.09%)	0.145
>6 g/day	1,128 (51.06%)	628 (49.68%)	500 (52.91%)	
Food diversity				
<4 types/day	1,042 (47.17%)	605 (47.86%)	437 (46.24%)	0.477
≥4 types/day	1,167 (52.83%)	659 (52.14%)	508 (53.76%)	
Hypertension				
No	1705 (77.18%)	1,053 (83.31%)	652 (68.99%)	<0.001
Yes	504 (22.82%)	211 (16.69%)	293 (31.01%)	
Hyperlipidemia				
No	1,290 (58.40%)	835 (66.06%)	455 (48.15%)	<0.001
Yes	919 (41.60%)	429 (33.94%)	490 (51.85%)	
Cardiovascular disease				
No	2,051 (92.85%)	1,169 (92.48%)	882 (93.33%)	0.495
Yes	158 (7.15%)	95 (7.52%)	63 (6.67%)	

Age is presented as mean ± standard deviation. Categorical variables are expressed as number (percentage).

### 3.2 Individual associations of tea consumption and cigarette smoking with accelerated biological aging

As shown in Table 2, tea drinking was positively associated with accelerated biological aging in the unadjusted model. Compared with non-tea drinkers, individuals who reported tea consumption had 63% higher odds of accelerated aging (*OR* = 1.63, 95% *CI*: 1.38–1.94, *P* < 0.001). After adjustment for demographic variables, the association was attenuated but remained significant (*OR* = 1.32, 95% *CI*: 1.10–1.58, *P* = 0.003). However, in the fully adjusted model, the association was no longer statistically significant (*OR* = 1.19, 95% *CI*: 0.99–1.45, *P* = 0.070).

**Table 2:** Individual effects of tea consumption and cigarette smoking on accelerated biological aging.

Variable	Category	Model 1		Model 2		Model 3	
		*OR* (95% *CI*)	*P*-value	*OR* (95% *CI*)	*P*-value	*OR* (95% *CI*)	*P*-value
**Tea drinking**	No	1.00 (Reference)		1.00 (Reference)		1.00 (Reference)	
	Yes	**1.63 (1.38, 1.94)**	**<0.001**	**1.32 (1.10, 1.58)**	**0.003**	1.19 (0.99, 1.45)	0.070
**Tea drinking level**	None	1.00 (Reference)		1.00 (Reference)		1.00 (Reference)	
	Low	**1.39 (1.12, 1.71)**	**0.002**	1.18 (0.95, 1.47)	0.138	1.10 (0.87, 1.38)	0.435
	High	**2.05 (1.65, 2.53)**	**<0.001**	**1.57 (1.25, 1.97)**	**<0.001**	**1.35 (1.06, 1.72)**	**0.016**
**Cigarette smoking**	No	1.00 (Reference)		1.00 (Reference)		1.00 (Reference)	
	Yes	**2.52 (2.12, 3.00)**	**<0.001**	**1.79 (1.43, 2.24)**	**<0.001**	**1.81 (1.42, 2.31)**	**<0.001**

Model 1, no covariate was adjusted. Model 2, adjusted for sex, ethnicity, education level, marital status, annual income. Model 3, further adjusted for body mass index, shift work, chemical substance exposure, noise exposure, dust exposure, alcohol drinking, physical activity, salt intake, food diversity, hypertension, hyperlipidemia, and cardiovascular disease. For tea drinking analyses, cigarette smoking was additionally adjusted in Model 3. For cigarette smoking analyses, tea drinking was additionally adjusted in Model 3. Results in bold indicate statistical significance. OR, odds ratio; CI, confidence interval.

When tea consumption was categorized by level, a clear dose-response trend was observed. Compared with non-tea drinkers, low-level tea drinkers had higher odds of accelerated aging in the unadjusted model (*OR* = 1.39, 95% *CI*: 1.12–1.71, *P* = 0.002), but this association was no longer significant after multivariable adjustment (Model 2: *OR* = 1.18, 95% *CI*: 0.95–1.47, *P* = 0.138; Model 3: *OR* = 1.10, 95% *CI*: 0.87–1.38, *P* = 0.435). In contrast, participants with high-level tea consumption consistently showed significantly increased odds of accelerated aging across all models. The unadjusted model showed a strong association (*OR* = 2.05, 95% *CI*: 1.65–2.53, *P* < 0.001), which remained significant after demographic adjustment (*OR* = 1.57, 95% CI: 1.25–1.97, *P* < 0.001) and even after full adjustment for all covariates (*OR* = 1.35, 95% CI: 1.06–1.72, *P* = 0.016).

For cigarette smoking, a robust and consistent association with accelerated biological aging was observed. Smokers had more than twice the odds of accelerated aging compared with non-smokers in the unadjusted model (*OR* = 2.52, 95% *CI*: 2.12–3.00, *P* < 0.001). Although adjustment for demographic variables attenuated the association, it remained highly significant (*OR* = 1.79, 95% *CI*: 1.43–2.24, *P* < 0.001). Further controlling for lifestyle, occupational, and clinical covariates did not materially alter the findings (*OR* = 1.81, 95% *CI*: 1.42–2.31, *P* < 0.001).

### 3.3 Combined effects of tea consumption and cigarette smoking on accelerated biological aging

**Figure 2:**
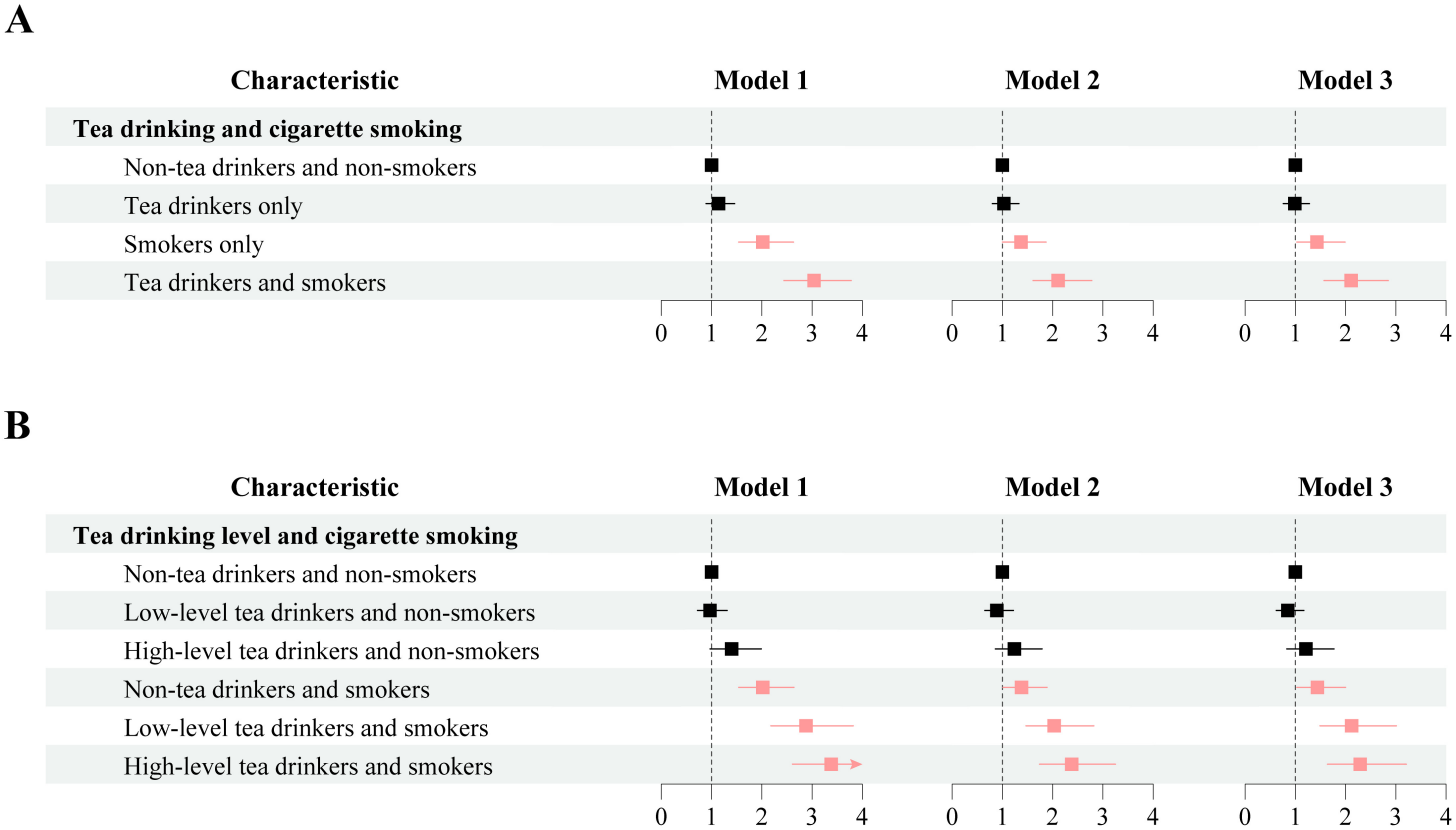
Combined effects of tea consumption and cigarette smoking on accelerated biological aging. **(A)** Tea drinking and cigarette smoking. **(B)** Tea drinking level and cigarette smoking. Model 1, no covariate was adjusted. Model 2, adjusted for sex, ethnicity, education level, marital status, and annual income. Model 3, further adjusted for body mass index, shift work, chemical substance exposure, noise exposure, dust exposure, alcohol drinking, physical activity, salt intake, food diversity, hypertension, hyperlipidemia, and cardiovascular disease. The color red represents statistical significance.

When tea consumption and cigarette smoking were considered jointly, distinct and graded associations with accelerated biological aging were observed (Supplementary Table 2). Compared with individuals who neither drank tea nor smoked, those who reported only tea drinking showed no significant difference in biological aging across all models. In contrast, participants who smoked but did not drink tea demonstrated significantly higher odds of accelerated aging, even after multivariable adjustment (Model 1: *OR* = 2.02, 95% *CI*: 1.54–2.63, *P* < 0.001; Model 2: *OR* = 1.37, 95% *CI*: 1.00–1.87, *P* = 0.047; Model 3: *OR* = 1.43, 95% *CI*: 1.03–1.99, *P* = 0.035). Notably, individuals who both smoked and drank tea exhibited the greatest risk (Figure 2A). Their odds of accelerated biological aging were more than tripled in the unadjusted model (*OR* = 3.04, 95% *CI*: 2.44–3.78, *P* < 0.001) and remained significantly elevated even after comprehensive covariate adjustment (*OR* = 2.11, 95% *CI*: 1.57–2.85, *P* < 0.001).

When tea consumption levels were further incorporated into the joint analysis, a clear dose-dependent gradient emerged (Figure 2B). Among non-smokers, neither low- nor high-level tea drinking was associated with accelerated aging after adjustment (low-level: *OR* = 0.85, 95% *CI*: 0.62–1.17, *P* = 0.324; high-level: *OR* = 1.21, 95% *CI*: 0.83–1.77, *P* = 0.323). However, among smokers, the risk of accelerated biological aging increased progressively with greater tea intake. Compared with individuals who neither drank tea nor smoked, those who smoked but did not drink tea exhibited a 44% higher likelihood of accelerated aging (*OR* = 1.44, 95% *CI*: 1.03–2.00, *P* = 0.031), while smokers with low-level tea drinking showed more than a twofold increase in the odds (*OR* = 2.12, 95% *CI*: 1.49–3.01, *P* < 0.001). Notably, participants who combined smoking with high-level tea consumption exhibited the greatest risk, with 129% higher odds of accelerated biological aging (*OR* = 2.29, 95% *CI*: 1.64–3.21, *P* < 0.001). Collectively, these findings indicate that increasing tea intake does not offset, but rather magnifies, the detrimental impact of cigarette smoking on biological aging.

In stratified analyses by smoking status, distinct patterns were observed (Supplementary Table 3). Among non-smokers, tea consumption was not significantly associated with accelerated biological aging, with no clear dose-response relationship across tea intake levels. In contrast, among smokers, higher tea consumption was associated with progressively increased odds of accelerated aging. Compared with smokers who did not consume tea, those with low-level tea intake had a 51% higher likelihood of accelerated aging (*OR* = 1.51, 95% *CI*: 1.08–2.13, *P* = 0.018), while those with high-level tea intake had a 65% higher likelihood (*OR* = 1.65, 95% *CI*: 1.19–2.29, *P* = 0.003). These findings suggest that the association between tea consumption and accelerated biological aging was confined to individuals with concurrent smoking exposure.

### 3.4 Interaction analyses

To quantify potential interactions, both additive and multiplicative scales were examined (Supplementary Table 4). A significant interaction between tea consumption and cigarette smoking was observed in relation to accelerated biological aging. On the additive scale, the combined exposure produced a greater-than-additive effect, with a *RERI* of 0.11, an *AP* of 0.09, and an *S* of 2.33, indicating that the joint effect was more than twice the sum of their individual effects. A significant multiplicative interaction was also detected (*P* = 0.025), further supporting a synergistic effect between tea consumption and cigarette smoking on biological aging. These findings suggest that tea drinking and cigarette smoking jointly contribute to the elevated risk, rather than acting independently.

### 3.5 Stratified analyses by shift work status

Stratified analyses were conducted to evaluate whether shift schedules modified the combined effects of tea drinking and cigarette smoking on accelerated biological aging (Table 3). Among non-shift workers, smoking alone was significantly associated with a higher likelihood of accelerated aging compared with the reference group of non-tea-drinking non-smokers (*OR* = 1.89, 95% *CI*: 1.04–3.44, *P* = 0.037). The combined exposure to both tea drinking and smoking showed an even stronger association (*OR* = 2.78, 95% *CI*: 1.62–4.81, *P* < 0.001). In contrast, these patterns were attenuated among shift workers, where smoking alone was not significantly related to accelerated aging, though the co-exposure to tea drinking and smoking remained significant (*OR* = 1.80, 95% *CI*: 1.25–2.59, *P* = 0.002). Further analyses stratified by tea consumption level revealed a consistent gradient among non-shift workers. Compared with the reference group, smokers who consumed low or high amounts of tea exhibited substantially greater risks (low-level: *OR* = 2.38, 95% *CI*: 1.25–4.62, *P* = 0.010; high-level: *OR* = 3.26, 95% *CI*: 1.77–6.09, *P* < 0.001). Similar patterns were observed among shift workers, although the magnitude of associations was comparatively smaller (low-level: *OR* = 1.88, 95% *CI*: 1.23–2.88, *P* = 0.004; high-level: *OR* = 1.91, 95% *CI*: 1.27–2.89, *P* = 0.002). Overall, the combined effects of tea consumption and cigarette smoking appeared more pronounced among non-shift workers, suggesting that factors beyond circadian disruption—such as baseline metabolic profiles or differential exposure to occupational hazards—may contribute to variation in susceptibility.

**Table 3:** Stratified analyses by shift work status for the combined effects of tea consumption and cigarette smoking on accelerated biological aging.

Variable	Non-shift work			Shift work		
	Number of participants	*OR* (95% *CI*)	*P*-value	Number of participants	*OR* (95% *CI*)	*P*-value
Tea drinking and cigarette smoking						
Non-tea drinkers and non-smokers	194	1.00 (Reference)		538	1.00 (Reference)	
Tea drinkers only	130	0.85 (0.50, 1.41)	0.524	334	1.03 (0.75, 1.41)	0.858
Smokers only	106	**1.89 (1.04, 3.44)**	**0.037**	223	1.19 (0.79, 1.79)	0.398
Tea drinkers and smokers	214	**2.78 (1.62, 4.81)**	**<0.001**	470	**1.80 (1.25, 2.59)**	**0.002**
Tea drinking level and cigarette smoking						
Non-tea drinkers and non-smokers	194	1.00 (Reference)		538	1.00 (Reference)	
Low-level tea drinkers and non-smokers	77	0.63 (0.32, 1.18)	0.155	208	0.93 (0.64, 1.35)	0.720
High-level tea drinkers and non-smokers	53	1.20 (0.57, 2.46)	0.629	126	1.20 (0.76, 1.89)	0.429
Non-tea drinkers and smokers	106	**1.89 (1.04, 3.45)**	**0.038**	223	1.20 (0.80, 1.80)	0.386
Low-level tea drinkers and smokers	92	**2.38 (1.25, 4.62)**	**0.010**	214	**1.88 (1.23, 2.88)**	**0.004**
High-level tea drinkers and smokers	122	**3.26 (1.77, 6.09)**	**<0.001**	256	**1.91 (1.27, 2.89)**	**0.002**

The models were adjusted for sex, ethnicity, education level, marital status, annual income, body mass index, chemical substance exposure, noise exposure, dust exposure, alcohol drinking, physical activity, salt intake, food diversity, hypertension, hyperlipidemia, and cardiovascular disease. Results in bold indicate statistical significance. OR, odds ratio; CI, confidence interval.

### 3.6 Sensitivity analyses

Several sensitivity analyses were conducted to verify the robustness of the main findings. First, when accelerated biological aging was analyzed as a continuous variable rather than a dichotomous outcome, the associations between tea consumption, cigarette smoking, and biological aging remained generally consistent in direction and magnitude (Supplementary Table 5). Second, when the outcome was reclassified based on the median value of biological aging as the cutoff, the results were materially unchanged (Supplementary Table 6). Third, after multiple imputation of missing covariates, the effect estimates for the combined exposure of tea consumption and cigarette smoking showed minimal changes compared with the primary results (Supplementary Table 7). Fourth, exclusion of participants with extreme biomarker values yielded similar results, further supporting the stability of the observed associations (Supplementary Table 8). Finally, using a refined classification of smoking status showed a comparable pattern of associations, with current smokers exhibiting significantly higher odds of accelerated biological aging, particularly when combined with tea consumption (Supplementary Table 9). Taken together, these analyses confirmed the robustness of our findings.

## 4 Discussion

In this occupational sample of oilfield workers, we observed that tea drinking was not significantly associated with accelerated biological aging after full covariate adjustment. When tea drinking levels were further categorized, a modest positive association emerged for individuals with higher tea intake, whereas low-level tea drinking showed no clear relationship with biological aging. In contrast, cigarette smoking exhibited a strong and consistent association with accelerated aging across all models, supporting its well-documented role in promoting age-related physiological deterioration. Notably, when tea consumption and smoking were evaluated together, participants who both smoked and drank tea—particularly at higher consumption levels—had markedly elevated odds of accelerated biological aging. The interaction analyses further indicated significant effects on both additive and multiplicative scales, suggesting that combined exposure to tea and tobacco may exert a synergistic impact on aging processes. These findings highlight the complex interplay between nutritional exposures and lifestyle factors in shaping biological aging trajectories.

The relationship between tea consumption and biological aging appears to be complex and context-dependent. In the present study, when analyzed independently, tea drinking did not demonstrate a clear protective association. After multivariable adjustment, however, individuals with high-level tea consumption showed a significant increase in the likelihood of accelerated biological aging, whereas the binary measure of tea drinking was not associated. Notably, this positive association was not observed among non-smokers in stratified analyses, indicating that tea consumption alone was not materially associated with accelerated aging in the absence of smoking exposure. This pattern suggests that the health implications of tea intake may depend not only on the amount consumed, but also on concurrent behavioral or environmental exposures, particularly cigarette smoking. Previous observational research has yielded heterogeneous conclusions. For example, prospective cohorts of Chinese adults reported that habitual tea consumption was associated with reduced frailty and slower biological aging trajectories (8, 9). In contrast, evidence on tea consumption and telomere-based aging markers is inconsistent, with one longitudinal study showing greater telomere loss among green tea drinkers and a Mendelian randomization analysis detecting no causal association between tea intake and telomere length (23, 24). Such discrepancies underscore the complexity of tea's physiological effects and suggest that its impact may vary according to individual characteristics and co-exposures. These results should therefore be interpreted with caution. One plausible explanation for the observed positive association with high-level tea consumption is reverse causation, whereby individuals experiencing greater fatigue, occupational stress, or early physiological decline may increase tea intake to maintain alertness and work performance. In this context, higher tea consumption may serve as a marker of underlying strain rather than a direct causal contributor to accelerated aging. Moreover, the absence of information on tea concentration, brewing practices, or recent changes in consumption limits causal inference and may partially account for this counterintuitive finding. In contrast, cigarette smoking exhibited a consistently strong association with accelerated biological aging, which aligns with extensive evidence linking tobacco exposure to molecular markers of aging. Numerous epigenetic studies have demonstrated that smokers exhibit greater acceleration of epigenetic age, as well as shorter leukocyte telomere length (15–17). Mechanistically, cigarette smoke induces persistent oxidative stress, promotes low-grade systemic inflammation, and disrupts protein homeostasis, all of which are key biological hallmarks of aging (13, 14). Our results reinforce these mechanistic insights by demonstrating that even after accounting for major demographic and health-related factors, smoking remains a dominant predictor of biological aging in this occupational sample.

When tea consumption and cigarette smoking were evaluated jointly, the combined exposure was associated with higher odds of accelerated biological aging compared with either exposure alone. In the joint exposure framework, individuals who both smoked and consumed tea exhibited substantially higher odds of accelerated biological aging compared with those who neither smoked nor drank tea. When tea consumption was further categorized by intake intensity, the risk increased in a dose-related manner among smokers, whereas no corresponding trend was observed among non-smokers. Participants who smoked and reported moderate tea intake had approximately twice the odds of accelerated aging, while those with high tea consumption showed an even greater likelihood. These stratified findings help mitigate concerns that the observed interaction merely reflects differences in smoking prevalence across tea consumption categories, and instead support an effect-modifying role of tea consumption within the smoking population. It is noteworthy that the association for the “smokers only” group among shift workers did not reach statistical significance, despite a directionally positive odds ratio. A more plausible explanation is that the elevated baseline levels of oxidative stress, circadian disruption, and metabolic strain associated with shift work may attenuate the observable main effect of smoking when considered in isolation. In this context, smoking-related risk may become more apparent when combined with additional lifestyle exposures, such as tea consumption, as reflected by the significant associations observed in the joint exposure categories. Quantitative interaction analyses further provided statistical evidence of interaction on both additive and multiplicative scales, although the magnitude of the additive interaction was modest. The measures of additive interaction revealed a relative excess risk due to interaction (*RERI* = 0.11), an attributable proportion (*AP* = 0.09), and a synergy index (*S* = 2.33), suggesting a positive departure from additivity at the population level. In addition, a significant multiplicative interaction was observed (*P* = 0.025), indicating that the joint association differed from that expected under a purely multiplicative model. Nevertheless, given the modest size of the additive interaction estimates, these results should be interpreted as evidence of statistical interaction rather than strong clinical amplification of risk attributable to tea consumption.

Stratified analyses by shift work status further revealed heterogeneity in the combined exposure effect. Among non-shift workers, smokers who also reported high-level tea consumption exhibited the highest likelihood of accelerated biological aging, with a clear gradient across increasing tea intake levels within the smoking group. In contrast, the combined effect was attenuated among shift workers. Notably, among shift workers who smoked, high-level tea consumption did not confer a substantially higher risk than low-level tea consumption, as reflected by similar odds ratios for the two joint exposure categories. This pattern suggests a potential ceiling effect, whereby the elevated baseline oxidative stress, circadian disruption, and metabolic strain associated with shift work may overshadow dose-related differences in tea intake within smokers. Alternatively, tea consumption among shift workers may differ in timing, context, or purpose, which could modify its interaction with smoking-related physiological stressors. Together, these findings indicate that work schedules may modulate the interplay between nutritional and behavioral exposures, underscoring the importance of considering occupational context when developing targeted strategies for biological aging prevention in working populations.

Mechanistically, this synergy may reflect converging pathways of oxidative stress, inflammation, and xenobiotic metabolism. Cigarette smoke generates persistent reactive oxygen species and promotes chronic inflammation, whereas tea polyphenols such as catechins modulate redox balance and cellular antioxidant defenses. In smokers, the oxidative burden from cigarette smoke may overwhelm tea-derived antioxidant activity or even shift certain polyphenols toward pro-oxidant effects (25, 26). Cigarette smoke also induces cytochrome P450 enzymes involved in xenobiotic metabolism, potentially altering the processing of tea-derived bioactive compounds (27, 28). In addition, while tea polyphenols can activate antioxidant pathways such as Nrf2–Keap1, chronic smoking may dysregulate this system and blunt adaptive protection, thereby amplifying oxidative and molecular damage (29, 30). Together, these results suggest that concurrent exposure to both behaviors may exert compounding biological stress, potentially accelerating molecular and systemic aging processes beyond the influence of either behavior alone.

From a public health perspective, these findings underscore the importance of integrated intervention strategies in occupational populations. While smoking cessation remains the primary target for reducing accelerated biological aging, nutritional behaviors such as tea consumption should be interpreted within the broader behavioral and occupational context, rather than in isolation. In high-risk working settings, coordinated programs that combine tobacco control, dietary counseling, and occupational health management may offer greater benefits than isolated behavior-specific interventions.

Despite the strengths of this study, several limitations should be acknowledged. First, the cross-sectional design precludes causal inference. Biological aging is inherently dynamic, yet accelerated aging in this study was inferred from a single time-point biomarker assessment. Such measurements may be influenced by short-term physiological fluctuations or early subclinical conditions and therefore cannot capture longitudinal aging trajectories. Second, tea consumption and smoking were self-reported, leading to potential misclassification. Moreover, tea consumption was assessed using a frequency-based questionnaire without information on tea type, brewing practices, or polyphenol content. Given the substantial variability in bioactive compounds across tea varieties and concentrations, this relatively coarse exposure assessment may have introduced non-differential misclassification and contributed to heterogeneity in the observed associations. Future studies incorporating more detailed dietary assessments and biomarkers of tea-derived polyphenols are warranted to clarify tea-specific effects on biological aging. Third, although a wide range of demographic, occupational, lifestyle, and clinical covariates were adjusted for, residual confounding cannot be entirely excluded. Unmeasured or incompletely characterized chronic conditions, such as autoimmune disorders or variation in disease severity, may still have influenced biomarker-based estimates of biological aging. Fourth, the study population consisted of oilfield workers in China, which may limit the generalizability of the findings to other occupational settings, age groups, or populations with different dietary and behavioral patterns. Finally, the absence of information on seasonal variation in tea consumption further introduces uncertainty in interpreting the exposure–aging relationship. Future studies with prospective designs, repeated biomarker measurements, and more refined assessments of tea consumption—including tea type, brewing strength, and objective biomarkers of tea-derived compounds—are warranted to clarify causal relationships and underlying biological mechanisms.

## 5 Conclusion

In conclusion, this study identifies a significant synergistic interaction between elevated levels of tea consumption and cigarette smoking in promoting accelerated biological aging among oilfield workers. Tea consumption alone was not significantly associated with accelerated aging, but its combination with smoking was linked to a markedly higher likelihood of accelerated aging. These findings underscore the need for targeted public health strategies and integrated nutritional and behavioral interventions to promote healthy aging in occupational settings.

## Data Availability

The raw data supporting the conclusions of this article will be made available by the authors, without undue reservation.
