# Supplementary material for: Combined associations of tea consumption and smoking with accelerated biological aging among oilfield workers
Source: Front Nutr. 2026 Jun 3;13:1762577. doi: 10.3389/fnut.2026.1762577 (PMC13272063; doi:10.3389/fnut.2026.1762577)
Supplement: Supplementary file 1 [file Data_Sheet_1.pdf]

Supplementary Material

Supplementary Table 1. Category and definition of covariates.

Supplementary Table 2. Combined effects of tea consumption and cigarette smoking on accelerated biological aging.

Supplementary Table 3. Association between tea consumption and accelerated biological aging stratified by smoking status.

Supplementary Table 4. Interaction effects of tea consumption and cigarette smoking on accelerated biological aging.

Supplementary Table 5. Combined effects of tea consumption and cigarette smoking on accelerated biological aging, treating phenotypic age acceleration as a continuous outcome.

Supplementary Table 6. Combined effects of tea consumption and cigarette smoking on accelerated biological aging, redefining the dichotomous cutoff using the median value.

Supplementary Table 7. Combined effects of tea consumption and cigarette smoking on accelerated biological aging, employing imputation for covariates.

Supplementary Table 8. Combined effects of tea consumption and cigarette smoking on accelerated biological aging, excluding participants with extreme biomarker values.

Supplementary Table 9. Combined effects of tea consumption and cigarette smoking on accelerated biological aging, using a refined classification of smoking status.

Supplementary Table 1. Category and definition of covariates.

Variable	Category and definition
Body mass index	Body mass index is calculated as weight in kilograms divided by height in meters squared (kg/m^2). Participants were classified into three categories: underweight/normal (<24 kg/m^2), overweight (24–27.9 kg/m^2), and obesity (≥28 kg/m^2).
Shift work	Shift work is defined as the regular rotation of individuals to work outside the hours of 8:00 AM to 5:00 PM for a minimum duration of one year.
Chemical substance exposure	Chemical substance exposure is defined as the self-reported exposure of individuals to hazardous chemical substances in the workplace or environment, including benzene, toluene, xylene, hydrogen sulfide, carbon monoxide, nitrogen oxides, carbon tetrachloride, n-hexane, n-pentane, gasoline, etc.
Noise exposure	Noise exposure is defined as the self-reported exposure of individuals to noise levels in the work environment that exceed legal noise standards.
Dust exposure	Dust exposure is defined as the self-reported exposure of individuals to inhalable or respirable dust particles during the work process, which may originate from production processes, material handling, or the surrounding environment.
Alcohol consumption	Alcohol consumption is defined as those who consume alcohol at least once a week and maintain this frequency for six months or longer.
Physical activity	Physical activity is assessed based on questionnaire items asking participants whether they usually engaged in moderate-intensity activities—such as slow jogging or moderate-paced cycling—lasting at least 10 minutes per session.
Salt intake	Salt intake is assessed using a questionnaire item asking participants about their usual daily salt consumption, with response options of ≤6 g/day and >6 g/day.
Food diversity	Food diversity is defined by the self-reported number of different food types consumed daily and categorized as <4 types/day and ≥4 types/day.
Hypertension	Hypertension is defined as systolic blood pressure ≥140 mmHg, diastolic blood pressure ≥90 mmHg, self-reported physician diagnosis of hypertension, or current use of antihypertensive medication.
Dyslipidemia	Dyslipidemia is defined as total cholesterol ≥6.2 mmol/L, triglycerides ≥2.3 mmol/L, low-density lipoprotein cholesterol ≥4.1 mmol/L, high-density lipoprotein cholesterol <1.0 mmol/L, self-reported physician diagnosis of hyperlipidemia, or current use of lipid-lowering medication.
Cardiovascular disease	Cardiovascular disease is defined as a self-reported history of coronary heart disease, atherosclerosis, or stroke.

Supplementary Table 2. Combined effects of tea consumption and cigarette smoking on accelerated biological aging.

Variable	Model 1		Model 2		Model 3	
	**OR (95 % CI)**	***P* value**	**OR (95 % CI)**	***P* value**	**OR (95 % CI)**	***P* value**
**Tea drinking and cigarette smoking**						
Non-tea drinkers and non-smokers	1.00 (Reference)		1.00 (Reference)		1.00 (Reference)	
Tea drinkers only	1.14 (0.89, 1.46)	0.307	1.03 (0.80, 1.33)	0.830	0.99 (0.76, 1.28)	0.914
Smokers only	**2.02 (1.54, 2.63)**	**<0.001**	**1.37 (1.00, 1.87)**	**0.047**	**1.43 (1.03, 1.99)**	**0.035**
Tea drinkers and smokers	**3.04 (2.44, 3.78)**	**<0.001**	**2.11 (1.61, 2.78)**	**<0.001**	**2.11 (1.57, 2.85)**	**<0.001**
**Tea drinking level and cigarette smoking**						
Non-tea drinkers and non-smokers	1.00 (Reference)		1.00 (Reference)		1.00 (Reference)	
Low-level tea drinkers and non-smokers	0.97 (0.72, 1.31)	0.858	0.89 (0.65, 1.22)	0.480	0.85 (0.62, 1.17)	0.324
High-level tea drinkers and non-smokers	1.40 (0.97, 1.99)	0.067	1.24 (0.86, 1.79)	0.250	1.21 (0.83, 1.77)	0.323
Non-tea drinkers and smokers	**2.02 (1.54, 2.64)**	**<0.001**	**1.38 (1.01, 1.89)**	**0.041**	**1.44 (1.03, 2.00)**	**0.031**
Low-level tea drinkers and smokers	**2.88 (2.18, 3.82)**	**<0.001**	**2.03 (1.47, 2.82)**	**<0.001**	**2.12 (1.49, 3.01)**	**<0.001**
High-level tea drinkers and smokers	**3.38 (2.60, 4.41)**	**<0.001**	**2.38 (1.74, 3.25)**	**<0.001**	**2.29 (1.64, 3.21)**	**<0.001**

Model 1, no covariate was adjusted. Model 2, adjusted for sex, ethnicity, education level, marital status, annual income. Model 3, adjusted for sex, ethnicity, education level, marital status, annual income, body mass index, shift work, chemical substance exposure, noise exposure, dust exposure, alcohol drinking, physical activity, salt intake, food diversity, hypertension, hyperlipidemia, and cardiovascular disease. Results in bold indicate statistical significance. OR, odds ratio; CI, confidence interval.

Supplementary Table 3. Association between tea consumption and accelerated biological aging stratified by smoking status.

Variable	Non-smoker		Smoker	
	**OR (95 % CI)**	***P* value**	**OR (95 % CI)**	***P* value**
**Tea drinking level**				
None	1.00 (Reference)		1.00 (Reference)	
Low	0.84 (0.61, 1.16)	0.299	**1.51 (1.08, 2.13)**	**0.018**
High	1.17 (0.79, 1.73)	0.422	**1.65 (1.19, 2.29)**	**0.003**

The models were adjusted for sex, ethnicity, education level, marital status, annual income, body mass index, shift work, chemical substance exposure, noise exposure, dust exposure, alcohol drinking, physical activity, salt intake, food diversity, hypertension, hyperlipidemia, and cardiovascular disease. Results in bold indicate statistical significance. OR, odds ratio; CI, confidence interval.

Supplementary Table 4. Interaction effects of tea consumption and cigarette smoking on accelerated biological aging.

Interaction scale	Interaction Indicator	Value
**Additive scale**		
	Relative Excess Risk of Interaction (RERI)	0.11
	Attributable Proportion (AP)	0.09
	Synergy Index (S)	2.33
**Multiplicative scale**		
	P value	0.025

The models were adjusted for sex, ethnicity, education level, marital status, annual income, body mass index, shift work, chemical substance exposure, noise exposure, dust exposure, alcohol drinking, physical activity, salt intake, food diversity, hypertension, hyperlipidemia, and cardiovascular disease.

Supplementary Table 5. Combined effects of tea consumption and cigarette smoking on accelerated biological aging, treating phenotypic age acceleration as a continuous outcome.

Variable	Model 1		Model 2		Model 3	
	***β* (95 % CI)**	***P* value**	***β* (95 % CI)**	***P* value**	***β* (95 % CI)**	***P* value**
**Tea drinking and cigarette smoking**						
Non-tea drinkers and non-smokers	0.00 (Reference)		0.00 (Reference)		0.00 (Reference)	
Tea drinkers only	0.028 (-0.028, 0.084)	0.319	0.006 (-0.051, 0.062)	0.846	-0.004 (-0.059, 0.052)	0.893
Smokers only	0.166 (0.103, 0.229)	**<0.001**	**0.076 (0.003, 0.148)**	**0.040**	**0.081 (0.008, 0.153)**	**0.029**
Tea drinkers and smokers	0.268 (0.217, 0.318)	**<0.001**	**0.182 (0.119, 0.245)**	**<0.001**	**0.172 (0.107, 0.236)**	**<0.001**
**Tea drinking level and cigarette smoking**						
Non-tea drinkers and non-smokers	0.00 (Reference)		0.00 (Reference)		0.00 (Reference)	
Low-level tea drinkers and non-smokers	-0.006 (-0.073, 0.062)	0.863	-0.024 (-0.092, 0.043)	0.484	-0.033 (-0.099, 0.033)	0.326
High-level tea drinkers and non-smokers	0.076 (-0.007, 0.158)	0.072	0.048 (-0.035, 0.131)	0.258	0.041 (-0.041, 0.123)	0.329
Non-tea drinkers and smokers	**0.166 (0.103, 0.229)**	**<0.001**	**0.078 (0.006, 0.151)**	**0.034**	**0.083 (0.010, 0.155)**	**0.026**
Low-level tea drinkers and smokers	**0.255 (0.189, 0.320)**	**<0.001**	**0.173 (0.097, 0.248)**	**<0.001**	**0.172 (0.095, 0.249)**	**<0.001**
High-level tea drinkers and smokers	**0.294 (0.233, 0.354)**	**<0.001**	**0.210 (0.139, 0.282)**	**<0.001**	**0.190 (0.117, 0.263)**	**<0.001**

Model 1, no covariate was adjusted. Model 2, adjusted for sex, ethnicity, education level, marital status, annual income. Model 3, adjusted for sex, ethnicity, education level, marital status, annual income, body mass index, shift work, chemical substance exposure, noise exposure, dust exposure, alcohol drinking, physical activity, salt intake, food diversity, hypertension, hyperlipidemia, and cardiovascular disease. Results in bold indicate statistical significance. CI, confidence interval.

Supplementary Table 6. Combined effects of tea consumption and cigarette smoking on accelerated biological aging, redefining the dichotomous cutoff using the median value.

Variable	Model 1		Model 2		Model 3	
	**OR (95 % CI)**	***P* value**	**OR (95 % CI)**	***P* value**	**OR (95 % CI)**	***P* value**
**Tea drinking and cigarette smoking**						
Non-tea drinkers and non-smokers	1.00 (Reference)		1.00 (Reference)		1.00 (Reference)	
Tea drinkers only	1.14 (0.90, 1.45)	0.274	1.01 (0.79, 1.29)	0.949	0.97 (0.75, 1.25)	0.803
Smokers only	**2.48 (1.90, 3.24)**	**<0.001**	**1.57 (1.15, 2.14)**	**0.004**	**1.68 (1.21, 2.34)**	**0.002**
Tea drinkers and smokers	**3.05 (2.46, 3.80)**	**<0.001**	**1.95 (1.49, 2.57)**	**<0.001**	**2.00 (1.49, 2.69)**	**<0.001**
**Tea drinking level and cigarette smoking**						
Non-tea drinkers and non-smokers	1.00 (Reference)		1.00 (Reference)		1.00 (Reference)	
Low-level tea drinkers and non-smokers	0.92 (0.69, 1.23)	0.593	0.83 (0.61, 1.12)	0.232	0.79 (0.58, 1.08)	0.140
High-level tea drinkers and non-smokers	**1.48 (1.05, 2.10)**	**0.026**	1.28 (0.90, 1.84)	0.172	1.27 (0.87, 1.84)	0.217
Non-tea drinkers and smokers	**2.48 (1.90, 3.24)**	**<0.001**	**1.59 (1.17, 2.17)**	**0.004**	**1.69 (1.22, 2.36)**	**0.002**
Low-level tea drinkers and smokers	**2.97 (2.24, 3.96)**	**<0.001**	**1.95 (1.40, 2.71)**	**<0.001**	**2.06 (1.45, 2.94)**	**<0.001**
High-level tea drinkers and smokers	**3.35 (2.57, 4.38)**	**<0.001**	**2.17 (1.59, 2.98)**	**<0.001**	**2.15 (1.53, 3.01)**	**<0.001**

Model 1, no covariate was adjusted. Model 2, adjusted for sex, ethnicity, education level, marital status, annual income. Model 3, adjusted for sex, ethnicity, education level, marital status, annual income, body mass index, shift work, chemical substance exposure, noise exposure, dust exposure, alcohol drinking, physical activity, salt intake, food diversity, hypertension, hyperlipidemia, and cardiovascular disease. Results in bold indicate statistical significance. OR, odds ratio; CI, confidence interval.

Supplementary Table 7. Combined effects of tea consumption and cigarette smoking on accelerated biological aging, employing imputation for covariates.

Variable	Model 1		Model 2		Model 3	
	**OR (95 % CI)**	***P* value**	**OR (95 % CI)**	***P* value**	**OR (95 % CI)**	***P* value**
**Tea drinking and cigarette smoking**						
Non-tea drinkers and non-smokers	1.00 (Reference)		1.00 (Reference)		1.00 (Reference)	
Tea drinkers only	1.06 (0.85, 1.32)	0.595	0.93 (0.74, 1.16)	0.507	0.88 (0.70, 1.11)	0.288
Smokers only	**2.13 (1.70, 2.68)**	**<0.001**	**1.33 (1.02, 1.74)**	**0.034**	**1.42 (1.07, 1.89)**	**0.014**
Tea drinkers and smokers	**2.68 (2.23, 3.24)**	**<0.001**	**1.72 (1.36, 2.18)**	**<0.001**	**1.74 (1.35, 2.24)**	**<0.001**
**Tea drinking level and cigarette smoking**						
Non-tea drinkers and non-smokers	1.00 (Reference)		1.00 (Reference)		1.00 (Reference)	
Low-level tea drinkers and non-smokers	0.94 (0.72, 1.21)	0.616	0.84 (0.64, 1.09)	0.201	0.80 (0.61, 1.05)	0.113
High-level tea drinkers and non-smokers	1.28 (0.95, 1.71)	0.105	1.08 (0.79, 1.46)	0.639	1.02 (0.74, 1.40)	0.897
Non-tea drinkers and smokers	**2.13 (1.70, 2.68)**	**<0.001**	**1.35 (1.03, 1.76)**	**0.030**	**1.43 (1.08, 1.90)**	**0.012**
Low-level tea drinkers and smokers	**2.54 (2.00, 3.21)**	**<0.001**	**1.65 (1.25, 2.18)**	**<0.001**	**1.74 (1.30, 2.34)**	**<0.001**
High-level tea drinkers and smokers	**2.80 (2.26, 3.47)**	**<0.001**	**1.80 (1.39, 2.34)**	**<0.001**	**1.76 (1.33, 2.32)**	**<0.001**

Model 1, no covariate was adjusted. Model 2, adjusted for sex, ethnicity, education level, marital status, annual income. Model 3, adjusted for sex, ethnicity, education level, marital status, annual income, body mass index, shift work, chemical substance exposure, noise exposure, dust exposure, alcohol drinking, physical activity, salt intake, food diversity, hypertension, hyperlipidemia, and cardiovascular disease. Results in bold indicate statistical significance. OR, odds ratio; CI, confidence interval.

Supplementary Table 8. Combined effects of tea consumption and cigarette smoking on accelerated biological aging, excluding participants with extreme biomarker values.

Variable	Model 1		Model 2		Model 3	
	OR (95 % CI)	*P* value	OR (95 % CI)	*P* value	OR (95 % CI)	*P* value
**Tea drinking and cigarette smoking**						
Non-tea drinkers and non-smokers	1.00 (Reference)		1.00 (Reference)		1.00 (Reference)	
Tea drinkers only	1.15 (0.89, 1.48)	0.288	0.98 (0.75, 1.28)	0.894	0.93 (0.71, 1.23)	0.629
Smokers only	**2.80 (2.12, 3.72)**	**<0.001**	**1.53 (1.11, 2.12)**	**0.010**	**1.61 (1.13, 2.28)**	**0.008**
Tea drinkers and smokers	**3.36 (2.67, 4.24)**	**<0.001**	**1.85 (1.39, 2.46)**	**<0.001**	**1.88 (1.37, 2.57)**	**<0.001**
**Tea drinking level and cigarette smoking**						
Non-tea drinkers and non-smokers	1.00 (Reference)		1.00 (Reference)		1.00 (Reference)	
Low-level tea drinkers and non-smokers	0.99 (0.73, 1.35)	0.964	0.87 (0.63, 1.20)	0.412	0.81 (0.58, 1.13)	0.216
High-level tea drinkers and non-smokers	1.40 (0.96, 2.03)	0.081	1.15 (0.78, 1.71)	0.478	1.16 (0.77, 1.75)	0.476
Non-tea drinkers and smokers	**2.80 (2.12, 3.72)**	**<0.001**	**1.55 (1.12, 2.14)**	**0.009**	**1.62 (1.14, 2.30)**	**0.007**
Low-level tea drinkers and smokers	**3.22 (2.39, 4.36)**	**<0.001**	**1.81 (1.28, 2.57)**	**<0.001**	**1.89 (1.30, 2.75)**	**<0.001**
High-level tea drinkers and smokers	**3.74 (2.83, 4.97)**	**<0.001**	**2.06 (1.48, 2.88)**	**<0.001**	**2.06 (1.44, 2.95)**	**<0.001**

Model 1, no covariate was adjusted. Model 2, adjusted for sex, ethnicity, education level, marital status, annual income. Model 3, adjusted for sex, ethnicity, education level, marital status, annual income, body mass index, shift work, chemical substance exposure, noise exposure, dust exposure, alcohol drinking, physical activity, salt intake, food diversity, hypertension, hyperlipidemia, and cardiovascular disease. Results in bold indicate statistical significance. OR, odds ratio; CI, confidence interval.

Supplementary Table 9. Combined effects of tea consumption and cigarette smoking on accelerated biological aging, using a refined classification of smoking status.

Variable	Model 1		Model 2		Model 3	
	OR (95 % CI)	*P* value	OR (95 % CI)	*P* value	OR (95 % CI)	*P* value
**Tea drinking and cigarette smoking**						
Non-tea drinkers and non-smokers	1.00 (Reference)		1.00 (Reference)		1.00 (Reference)	
Non-tea drinkers and former smokers	1.57 (0.89, 2.73)	0.114	1.10 (0.61, 1.97)	0.742	1.12 (0.60, 2.07)	0.714
Non-tea drinkers and current smokers	**2.12 (1.60, 2.82)**	**<0.001**	**1.43 (1.03, 1.98)**	**0.032**	**1.49 (1.05, 2.10)**	**0.025**
Tea drinkers and non-smokers	1.14 (0.89, 1.46)	0.307	1.03 (0.80, 1.33)	0.833	0.98 (0.75, 1.28)	0.898
Tea drinkers and former smokers	1.43 (0.94, 2.17)	0.094	1.02 (0.64, 1.60)	0.943	0.96 (0.59, 1.55)	0.864
Tea drinkers and current smokers	**3.50 (2.79, 4.41)**	**<0.001**	**2.42 (1.82, 3.21)**	**<0.001**	**2.43 (1.79, 3.30)**	**<0.001**
**Tea drinking level and cigarette smoking**						
Non-tea drinkers and non-smokers	1.00 (Reference)		1.00 (Reference)		1.00 (Reference)	
Low-level tea drinkers and non-smokers	0.97 (0.72, 1.32)	0.858	0.89 (0.65, 1.22)	0.478	0.85 (0.61, 1.17)	0.314
High-level tea drinkers and non-smokers	1.40 (0.97, 1.99)	0.067	1.24 (0.85, 1.78)	0.256	1.20 (0.82, 1.76)	0.340
Non-tea drinkers and former smokers	1.57 (0.89, 2.73)	0.115	1.11 (0.61, 1.99)	0.717	1.13 (0.61, 2.08)	0.696
Low-level tea drinkers and former smokers	1.13 (0.57, 2.17)	0.716	0.82 (0.40, 1.61)	0.568	0.79 (0.37, 1.59)	0.511
High-level tea drinkers and former smokers	1.75 (0.99, 3.05)	0.051	1.24 (0.68, 2.23)	0.479	1.08 (0.58, 2.01)	0.812
Non-tea drinkers and current smokers	**2.12 (1.60, 2.82)**	**<0.001**	**1.44 (1.04, 2.00)**	**0.029**	**1.49 (1.06, 2.11)**	**0.023**
Low-level tea drinkers and current smokers	**3.36 (2.50, 4.55)**	**<0.001**	**2.35 (1.68, 3.32)**	**<0.001**	**2.47 (1.71, 3.56)**	**<0.001**
High-level tea drinkers and current smokers	**3.82 (2.89, 5.07)**	**<0.001**	**2.67 (1.92, 3.70)**	**<0.001**	**2.59 (1.83, 3.68)**	**<0.001**

Smoking status was classified into three categories. Never smokers were defined as individuals who had never smoked cigarettes. Former smokers were defined as those who had smoked in the past but had completely quit smoking at the time of the survey. Current smokers were defined as individuals who smoked at least one cigarette per day for six months or longer and continued smoking at the time of the survey. Model 1, no covariate was adjusted. Model 2, adjusted for sex, ethnicity, education level, marital status, annual income. Model 3, adjusted for sex, ethnicity, education level, marital status, annual income, body mass index, shift work, chemical substance exposure, noise exposure, dust exposure, alcohol drinking, physical activity, salt intake, food diversity, hypertension, hyperlipidemia, and cardiovascular disease. Results in bold indicate statistical significance. OR, odds ratio; CI, confidence interval.
